# The Mutual Contribution of 3-NT, IL-18, Albumin, and Phosphate Foreshadows Death of Hemodialyzed Patients in a 2-Year Follow-Up

**DOI:** 10.3390/antiox11020355

**Published:** 2022-02-11

**Authors:** Łukasz Kasprzak, Mateusz Twardawa, Piotr Formanowicz, Dorota Formanowicz

**Affiliations:** 1 Department of Nephrology with Dialysis Unit, Provincial Hospital in Leszno, 64-100 Leszno, Poland; lukakas@wp.pl; 2 Institute of Computing Science, Poznan University of Technology, 60-965 Poznan, Poland; Mateusz.Twardawa@cs.put.poznan.pl (M.T.); Piotr.Formanowicz@cs.put.poznan.pl (P.F.); 3 ICT Security Department, Poznan Supercomputing and Networking Center Affiliated to the Institute of Bioorganic Chemistry, Polish Academy of Sciences, 61-139 Poznan, Poland; 4 Institute of Bioorganic Chemistry, Polish Academy of Sciences, 61-704 Poznan, Poland; 5 Department of Medical Chemistry and Laboratory Medicine, Poznan University of Medical Sciences, 60-806 Poznan, Poland

**Keywords:** hemodialysis, mortality, inflammation, nitrosative stress, phosphate, IL-18, predictive model

## Abstract

Patients with chronic kidney disease (CKD), especially those who are hemodialyzed (HD), are at significantly high risk of contracting cardiovascular disease and having increased mortality. This study aimed to find potential death predictors, the measurement of which may reflect increased mortality in HD patients, and then combine the most promising ones in frames of a simple death risk assessment model. For this purpose, HD patients (n=71) with acute myocardial infarction in the last year (HD group) and healthy people (control group) as a comparative group (n=32) were included in the study. Various laboratory determinations and non-invasive cardiovascular tests were performed. Next, patients were followed for two years, and data on cardiovascular (CV) deaths were collected. On this basis, two HD groups were formed: patients who survived (HD-A, n=51) and patients who died (HD-D, n=20). To model HD mortality, 21 out of 90 potential variables collected or calculated from the raw data were selected. The best explanatory power (95.5%) was reached by a general linear model with four variables: interleukin 18, 3-nitrotyrosine, albumin, and phosphate. The interplay between immuno-inflammatory processes, nitrosative and oxidative stress, malnutrition, and calcium-phosphate disorders has been indicated to be essential in predicting CV-related mortality in studied HD patients. ClinicalTrials.gov Identifier: NCT05214872.

## 1. Introduction

Chronic kidney disease (CKD), defined as a progressive loss of functional nephrons leading to a gradual reduction in the glomerular filtration rate (GFR) and increased urinary albumin excretion, has become a global public health problem [1] and a recognized risk factor for cardiovascular disease (CVD) [2].

Epidemiological studies have shown that in 2017, the global incidence of CKD was 9.1%. Whereas it increased in all age groups by 29.3% between 1990 and 2017, the age-standardized global incidence has not changed significantly. CKD caused 1.2 million deaths in 2017 and has been found to be the 12th most common cause of death globally. Moreover, 7.6% of all CVD deaths have been linked to impaired kidney function. Overall, deaths from CKD or CVD attributed to CKD have accounted for 4.6% of all deaths. Between 1990 and 2017, global mortality from CKD at all ages increased by 41.5%. In contrast, age-standardized CKD mortality has remained stable, contrary to the declined age-standardized mortality worldwide for CVD, cancer, and chronic obstructive pulmonary disease [3].

It has been proven that the incidence and severity of CVD increases with a decrease in the GFR. Coronary artery disease (CAD) in CKD has shown a pattern of diffuse multi-vessel involvement with calcification in the coronary arteries in kidney failure patients [4]. In the cohort studies, half of all patients with advanced CKD (stages 4 and 5) had suffered from CVD, and cardiovascular (CV)-related mortality accounted for 40–50% of all their deaths as compared with 26% among healthy, age-matched controls [1,5]. Additionally, in 25- to 34-year-old patients with kidney failure, annual mortality was found to have increased from 500- to 1000-fold, which corresponded to that of 85-year-olds in the general population [1].

In the face of such facts, special attention has been paid to understanding the mechanisms leading to the worsening of CVD in CKD and the increasing mortality rate in CKD.

This topic has turned out to be challenging because of the multifaceted nature of the disorders in which the kidney is involved. Accumulating evidence has suggested that the kidneys are not only the target organs of many diseases, but that they can strikingly exacerbate or trigger systemic patho-physiological processes through their complex functions and influence the homeostasis of the whole organism. Due to its essential role in circulation, the kidney has often been revealed to be a target organ for systemic vascular, hemodynamic, metabolic, and inflammatory disorders. The involvement of the kidneys in many pathological processes of non-renal origin has been found to worsen CKD as well as the prognosis [6].

Traditional CV risk factors have been found to be very commonly observed in CKD patients, and their contribution to CVD has been revealed to be especially important in the early CKD stages. Hypertension, dyslipidemia, insulin resistance, diabetes, and smoking, due to their impact on the large and smaller renal vessels, have been revealed to affect both the CV and cerebrovascular system, but also the progression of CKD [7]. Hypertension and CKD have been proven to be closely related conditions so that persistent hypertension can lead to the worsening of renal function, and progressive renal impairment may conversely lead to the deterioration of blood pressure control [8]. Studies have shown that hypertension is present in 70% of CKD patients and 80–90% of kidney failure patients [9]. On the other hand, hypertension and other traditionally recognized risk factors such as advanced age, male gender, tobacco smoking, and the increased concentration of total cholesterol in the blood serum have failed to thoroughly explain the high incidence of CVD and CV-related mortality in CKD patients.

The unique nature of CVD in CKD is constantly suggested [10,11,12,13]. It has been postulated that classical and non-classical risk factors are most likely involved in CVD progression among CKD patients in different proportions, and their significance is changing due to the progressive nature of CKD. The more advanced the renal dysfunction is, the less significant these so-called classical CV risk factors are. On the other hand, it has been shown to be unreasonable to unequivocally omit the significance of classical CV risk factors. However, it should be noted that the multi-threading CVD aspects in CKD may lead to paradoxical conclusions. In dialyzed patients with CKD, an inverse relationship between serum cholesterol levels and mortality, known as “reverse epidemiology”, has been reported [14,15]. It has been explained that several factors, including malnutrition and systemic inflammation [11], may cause the inverse relationship between cholesterol levels and mortality.

Apart from the role of the traditional risk factors, two primary mechanisms have been distinguished as contributing to CVD development in CKD. The first mechanism is directly based on the kidney action, which may release hormones, cytokines, and enzymes in response to injury or failure, thus contributing to the characteristic vascular changes. The second mechanism has relied on CKD mediators and hemodynamic changes leading to cardiac damage [16]. Furthermore, it has been revealed that many abnormalities accompany the loss of the excretory function of the kidneys, such as those in the water and electrolyte levels, the acid–base, redox, and endocrine systems, and in the circulation and regulation of the renal–bone–vascular pathways. Disturbances in the internal homeostasis lead to the irreversible modification of proteins, lipids, carbohydrates, and nucleic acids, both due to the disruption of the metabolic pathways and the formation of pathological compounds of high reactivity that react with other molecules. In the mechanism of positive feedback, a change in the structure and action of critical molecules for the proper functioning of cells and organs has been revealed, causing further progression of CKD and the development of systemic complications [5].

Much research has been performed to date to find variables that could be used to predict CV mortality in hemodialyzed (HD) patients. Many potential markers have been indicated. However, so far, none of these are strong enough and could be recommended. Recently, Hansrivijit et al., in their systematic review, indicated a total of seven predictive factors as excellent: N-terminal pro-brain natriuretic peptide (NT-proBNP), BNP, soluble urokinase plasminogen activator receptor (suPAR), augmentation index, left atrial reservoir strain, C-reactive protein (CRP), and systolic pulmonary artery pressure. Another seventeen predictive factors were found to be within acceptable ranges, and the authors classified them into the following subgroups: predictors for the non-dialysis population, echocardiographic factors, comorbidities, and miscellaneous [17]. It shows how complex the issue of CV mortality in HD patients must be when there are so many different factors to consider.

Our study aimed to create a model for assessing the risk of death in HD patients with a history of acute myocardial infarction (AMI) in the previous year, based on a 2-year follow-up. HD patients were tested to identify potential markers; their collective assessment could reflect the mortality observed in this patient group.

## 2. Materials and Methods

### 2.1. Study Group

A total of 103 people were enrolled in the study, including 71 consecutive HD patients with AMI in the last year (HD group) and treated in 2016–2018 at the Nephrology Outpatient Clinic and the Clinical Hospital’s Dialysis Center, H. Święcickiego in Poznań; 32 healthy participants (control group) served as a comparative group.

In the HD group, the duration of the HD sessions was at least 12 h/week; standard bicarbonate dialysis fluids and polysulfone low-flux dialyzers were used. HD procedures were performed on each patient three times a week via an arteriovenous fistula from their own or artificial vessels. The blood flow during hemodialysis was 200–350 mL/min, with an average dialysis fluid flow of 500 mL/min. The most common cause of the kidney failure was hypertensive nephropathy (n=54), while in the remaining patients it was vasculitis (n=8) or chronic glomerulonephritis (n=9). The most commonly used drugs were loop diuretics, angiotensin-converting enzyme inhibitors, angiotensin II receptor antagonists, calcium channel blockers, centrally acting antihypertensive drugs (clonidine hydrochloride, methyldopa), alpha-blockers, calcium binders (calcium carbonate or calcium acetate), and vitamin D and its derivatives (alfacalcidol). Some patients received erythropoiesis-stimulating agents (ESA) by vein injection.

During a 2-year follow-up from the enrollment to this study, a history of CV-related fatal incidents was separately recorded for each subject. The primary endpoint was fatal AMI or acute ischemic stroke or any unexpected or sudden death, but only if autopsy proved it to be CV-related. If there was doubt about the cause of death or there was no contact with the patient during the two years from study enrollment, those patients were excluded and not considered further. Based on the achievement of the endpoint, HD patients were divided into two subgroups: living (HD-A, n=51) and patients who died during the two-year survey (HD-D, n=20).

A control group was recruited from subjects with no clinical and laboratory evidence of renal and cardiovascular dysfunction at study entry and no positive medical history. These were people recruited from those reporting to the laboratory for routine checkups.

The exclusion criteria for both groups were: active acute infection, immunosuppressive therapy, kidney transplant, abnormal liver function, malignant tumors within the last five years, and alcohol abuse within the last five years.

### 2.2. Basic Assessments

Extended blood test results and standard metrical and medical information were collected for both studied groups.

Blood samples were taken from all fasting subjects during the same period of the day, between 8:00 and 9:00. The sampling procedure was identical for all groups tested. Blood collection from dialysis patients had to be synchronized with the dialysis schedule. Blood samples from HD patients were drawn in the morning (prior to heparin administration) prior to the second HD in the week of scheduled routine blood tests.

All samples were stored at −80 ${}^{\circ }$C and processed in the same way. Analyses of individual parameters were carried out constantly by the same people in similar weather conditions.

All assessed laboratory parameters were performed using standard methods, in accordance with the manufacturer’s instructions. For more detailed information, concerning methods that were used, see ClinicalTrials.gov Identifier: NCT05214872.

The following parameters were determined in all studied groups:

- the complete blood count, glucose (Glu), total protein (TP), albumin (ALB), creatinine, urea; parameters of lipid metabolism: total cholesterol, low-density lipoprotein cholesterol (LDL-C), high-density lipoprotein cholesterol (HDL-C), triglycerides (TG), potasium (K), sodium (Na), magnesium (Mg); parameters of iron metabolism: iron concentration, total iron binding capacity (TIBC), the unsaturated iron binding capacity (UIBC), and ferritin concentration; activity of alanine transaminase (ALT), aspartate transaminase (AST), and alkaline phosphatase (ALP); parameters of calcium and phosphate metabolism: total and ionized calcium, phosphate (PO${}_{4}^{3-}$), intact parathormone (iPTH), and high-sensitivity C-reactive protein (hsCRP) were assessed by routine techniques;
- estimated GFR (eGFR)—according to the KDIGO 2012 recommendations—was calculated based on the Modification of Diet in Renal Disease (MDRD) formula: eGFR = 186 × (creatinine concentration (mg/dL)) − 1.154 × (age) − 0.203 × (0.724) for the female gender;
- serum concentration of the selected inflammatory markers: neopterin and interleukin 18 (IL-18); oxidative stress parameters: advanced oxidation protein products (AOPP), advanced glycation ends products (AGE), carboxymethyl${}^{\epsilon }$(lysine) (CML) and 3-nitrotyrosine (3NT), carbonyl groups of proteins, methylglyoxal (MG), carboxyethyl${}^{\epsilon }$(lysine) (CEL) and carbamyl groups of proteins (CBL-BSA), soluble receptor for advanced glycation end products (sRAGE) and myeloperoxidase (MPO), klotho (KL), fibroblast growth factor 23 (FGF-23); metalloproteinases: metalloproteinase 9 (MMP-9), tissue inhibitor of metalloproteinase 1 (TIMP-1), and NT-pro-brain natriuretic peptide (NT-proBNP) were determined by the enzyme-linked immunosorbent assay (ELISA) method using appropriate kits;
- body mass index (BMI) (kg/m${}^{2}$) was calculated by dividing a person’s weight (post-HD weight in the HD group) (kg) by the square of their body height (m);
- carotid intima-media thickness (IMT) was measured by The Accuson CV 70 system (Siemens) with a 10 MHZ transducer. Two longitudinal projections were assessed (antero-lateral and postero-lateral). The distal 1cm of the common carotid artery just proximal to the bulb was measured by means of a computer analysis system (Medical Imaging Applications, LLC).

For non-invasive cardiological examinations, the Portapres TM device (FMS, Enschede, The Netherlands), Pulse Trace 2000TM (Micro Medical Ltd., Rochester, Kent, UK), the SphygmoCor MxTM tonometer (ATCOR, Naperville, IL, USA), the Colin CBM-7000 TM device (Colin Medical Instruments Corp., San Antonio, TX, USA), and the Acuson CV 70 ultrasound machine (Siemens, Erlangen, Germany) were used. The system was equipped with a vascular head operating at 10 MHz.

The evaluation of the variables using the non-invasive devices was based on advanced algorithms for analyzing pulse waveforms and EKG curves. The SphygmoCor system (ATCOR, Naperville, IL, USA) used derived the central aortic pressure waveform from cuff pulsations recorded at the brachial artery. Analysis of the waveforms provided the values of the key parameters, including the central systolic pressure (cSP), central pulse pressure (cPP), and indices of arterial stiffness such as augmentation pressure (cAP) and augmentation index (cAI). Moreover, Pulse Trace TM enabled the assessment of the following: reflection index (RI), vascular stiffness index (SI), peripheral pulse pressure (pPP), and peripheral pulse pressure/central pulse pressure (pPP/cPP ratio). A more detailed explanation of the methods of determining the individual variables can be found at https://atcormedical.com/technology/sphygmocor/ (accessed on 7 December 2021). The values of the parameters that were taken into account in order to find the best predictive model are included in the Supplementary Materials.

### 2.3. Ethics Statement

The study was carried out in accordance with the Declaration of Helsinki of the World Medical Association and approved by the Bioethics Committee at Poznan University of Medical Sciences (decision No. 274/16 of 3 March 2016). All included study participants fulfilled the criteria and completed the study. They were fully informed about the study, and all of them gave written informed consent before the enrollment.

### 2.4. Statistical Analysis and Modeling

In order to find factors associated with death risk, exploratory statistical analysis was performed. Based on the obtained results and the current state of knowledge, variables for building and testing the generalized linear model (GLM) were selected. The performed statistical analysis and model building can be divided into the following steps:

1. Detecting the most important differences in variable levels between hemodialyzed patients who died and those who survived within two years of follow-up;
2. Checking whether differences found are unique to deceased hemodialyzed patients or if distortions are specific for all hemodialyzed subjects by comparing to the control group;
3. Selecting the most strongly differentiating variables found in the previous steps;
4. Finding significant and strong correlations within selected variables for hemodialyzed patients;
5. Model building and testing;
6. Model diagnostics and results analysis.

All steps were performed on Python 3.8. The following packages were used to execute statistical analysis: numpy-1.19.5, pandas-1.2.4, statmodels-0.12.2, scipy-1.3.3. Data and results were visualized on Python with the help of the matplotlib-3.1.2 and seaborn-0.11.0 packages.

Basic information concerning the analyzed groups of patients is presented in Table 1. For detection of dissimilarities between groups (HD-D vs. HD-A, Control vs. HD-D, Control vs. HD-A), the Kruskal–Wallis H test was used (based on Shapiro–Wilk test results, it was concluded that the assumptions needed to run parametric tests were not satisfied in almost all cases). Results for the Kruskal–Wallis H test are summarized in Table 2 and Table 3. Only significantly different variables were selected, and for this subset, a correlative structure of the data was analyzed. To do so, Spearman rank correlation tests were used. Moreover, correlations for data inside the HD group and the control group were separately visualized (see Figure S1 in Supplementary Materials). Significant correlations for HD patients are presented in Table S1 in the Supplementary Materials. Based on the obtained results, the most promising variables to create GLM with a binomial distribution that would predict mortality within two years of follow-up were selected. Predictor selection for the final model was based on the Kruskal–Wallis H test results, Spearman correlation results, current medical knowledge, and the forward stepwise selection [18] process of fitting selected variables to the model. At the last stage, the model residuals were analyzed. The residual diagnostics made it possible to check residual distribution and detect outliers.

## 3. Results

### 3.1. General Description of the Study Group

Basic information about the analyzed patients are summarized in Table 1. The mean age of the patients did not differ between the hemodialyzed patients and the control group. In contrast, the mean age of the HD-D group was approximately 10 years higher than that of the HD-A group, and this difference is significant (see Table 3). Females were less represented than males in the HD group (1/3 of patients). Moreover, there were more smokers in the HD-A group and less overweight patients (BMI > 25 kg/m${}^{2}$ was considered as overweight). Patients forming the HD-D showed an eGFR slightly higher than the HD-A, and the urea serum concentration was lower in the HD-D vs. HD-A. hsCRP, a marker of the low-grade inflammatory process, was higher among the HD-D group compared to the HD-A group. What is interesting is that the mean duration of the HD treatment was much shorter in the HD-D group than in the HD-A. Hemodialyzed patients in the HD-D group lived, on average, for almost one year after enrollment in the study.

The results of all performed analyses and comparisons between HD-D and HD-A, HD-D and control, and HD-A and the control group are included in the Supplementary Materials.

### 3.2. Differences between the Groups

**Figure 1:**
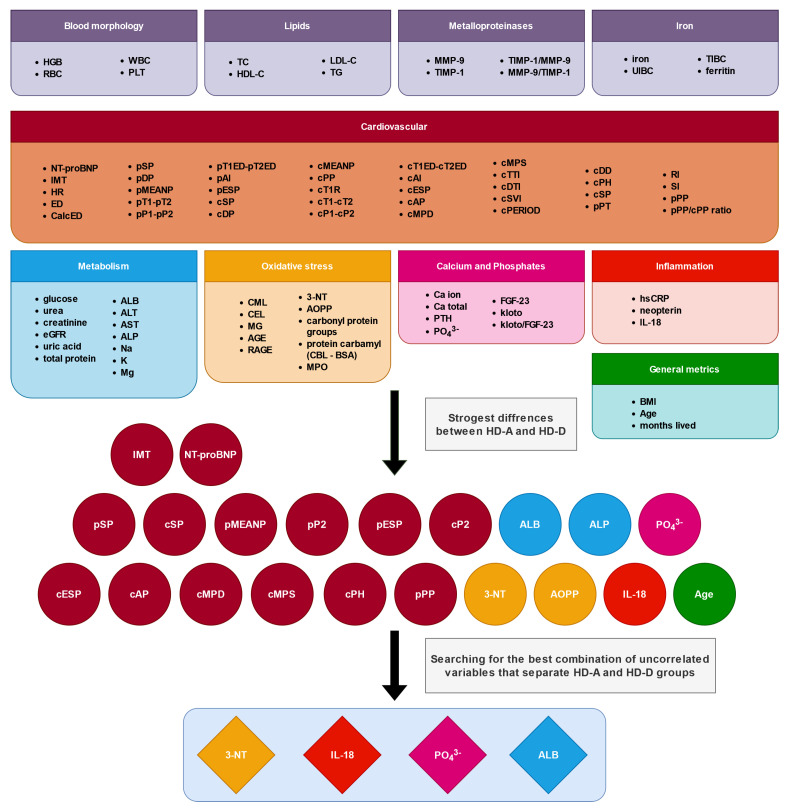
Stages of variable selection for the creation of the final model. We have distinguished 10 categories of variables: blood morphology, lipids, metalloproteinases, iron, cardiovascular, metabolism, oxidative stress, calcium and phosphates, inflammation, and general metrics. Four categories ware completely rejected based on the Kruskal–Wallis test results. Spearman correlation and experimental model fitting helped to find the best combination of variables that differentiate the HD-A and HD-D groups (4 out of 21).

The full list of variables along with statistical selection flow is presented in Figure 1. Among the analyzed variables, a few that strongly differ between the HD-D and HD-A groups were detected—they are shown in Table 2 and Table 3. Although, hemodialyzed patients show multiple and often huge differences in comparison to healthy people according to Table 2 and Table 3, not all of them can be attributed simply to kidney malfunction and treatment. The presented pairwise comparison reveals differences that are unique for patients who died (e.g., ALP and IMT). A similar situation is found for the following cardiovascular parameters: pESP, cESP, cAP and cMPD.

The strongest and most significant differences have been found for albumin, 3-NT, IL-18, AOPP, NT-proBNP, and phosphates, which may suggest the importance of kidney failure-associated factors such us inflammation, malnutrition, and nitrosative and oxidative stress.

**Figure 2:**
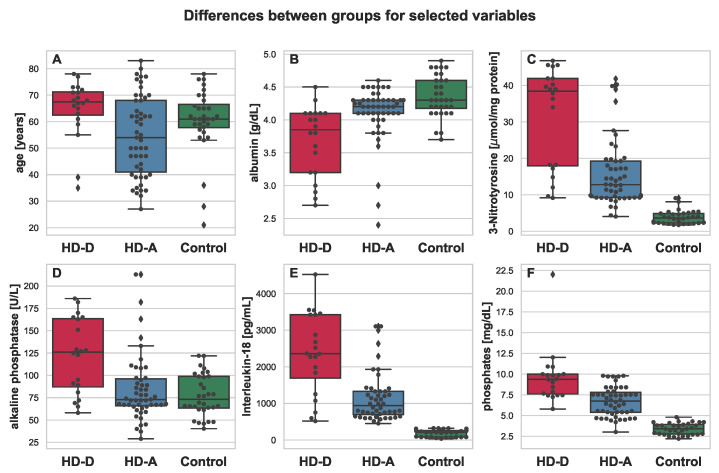
Boxplot depicting differences between HD-D (red), HD-A (blue), and Control (green) for the chosen variables: age (**A**), albumin (**B**), 3-Nitrotyrosine (**C**), alkaline phosphatase (**D**), Interleukin-18 (**E**), and phosphates (**F**). Additional swarmplot was overlaid onto the original plot in order to mark the obtained measurements for each of the patients (dots). Outlier values are represented by diamons.

**Figure 3:**
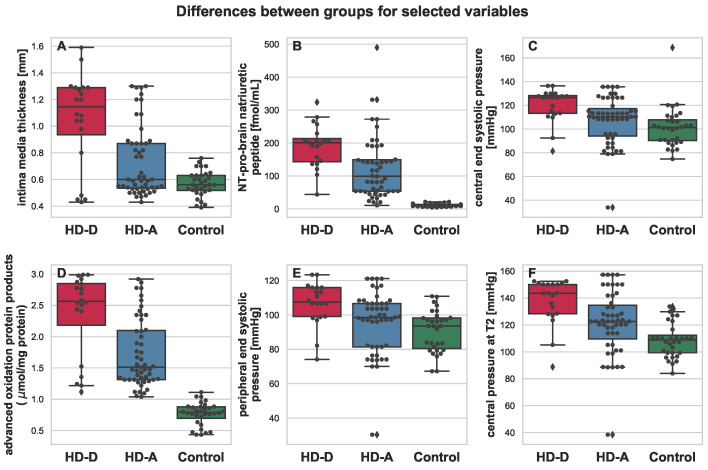
Boxplot depicting differences between HD-D (red), HD-A (blue), and Control (green) for the chosen variables: intima media thickness (**A**), NT-pro-brain natriuretic peptide (**B**), central end systolic pressure (**C**), advanced oxidation protein products (**D**), peripheral end systolic pressure (**E**), and central pressure at T2 (**F**). Additional swarmplot was overlaid onto the original plot in order to mark the obtained measurements for each of the patients (dots). Outlier values are represented by diamons.

**Figure 4:**
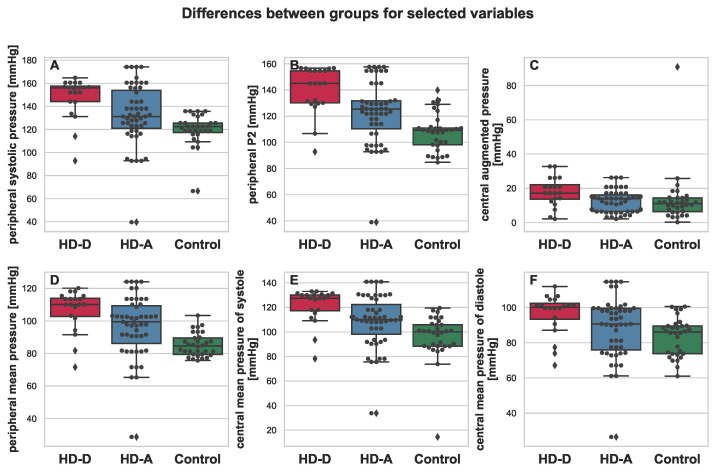
Boxplot depicting differences between HD-D (red), HD-A (blue), and Control (green) for the chosen variables: peripheral systolic pressure (**A**), peripheral P2 (**B**), central augmented pressure (**C**), peripheral mean pressure (**D**), central mean pressure of systole (**E**), and central mean pressure of diastole (**F**). Additional swarmplot was overlaid onto the original plot in order to mark the obtained measurements for each of the patients (dots). Outlier values are represented by diamons.

**Figure 5:**
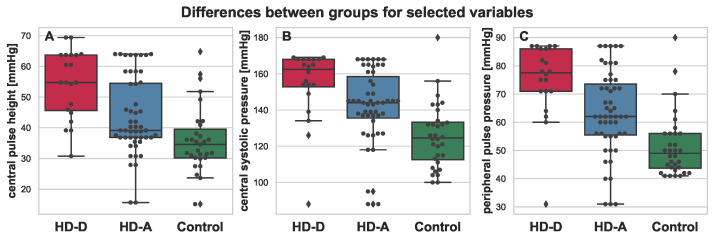
Boxplot depicting differences between HD-D (red), HD-A (blue), and Control (green) for the chosen variables: central pulse height (**A**), central systolic pressure (**B**), and peripheral pulse pressure (**C**). Additional swarmplot was overlaid onto the original plot in order to mark the obtained measurements for each of the patients (dots). Outlier values are represented by diamons.

All mentioned parameters, except for albumin, were elevated in the HD-A and HD-D groups. It is also worth noting that for almost every parameter, much higher variance was present in the group of HD patients (see Figure 2, Figure 3, Figure 4 and Figure 5). This was indeed a good premise to combine multiple variables in order to differentiate the HD-A and HD-D groups.

### 3.3. Correlations within the Groups

The correlation between variables selected in the previous step helps to evaluate them as potential death predictors, especially if taken in mutual complementation (see Table S1 and Figure S1 in Supplementary Materials). All chosen cardiovascular parameters were highly correlated, which was anticipated. In addition, only two other significant correlations were found, i.e., 3-NT with AOPP and pP2 with IL-18.

### 3.4. GLM for Mortality within 2 Years of Follow-Up

The best GLM model to differentiate patients who died from those who survived relied on 4 variables: Interleukin-18, 3-NT, ALB, and phosphates. At this stage, it was necessary to exclude 4 patients belonging to the HD-A group due to a lack of IL-18 (3 patients) or phosphates (1 patient) measurements assigned to them in the dataset. The model can be summarized with the following equation:

(1) $$\begin{array}{c} Y=1.9160+\left(0.1314\cdot 3\text{-}\mathrm{NT}\left[\frac{\mu \mathrm{mol}}{\mathrm{mg}\;\mathrm{protein}}\right]\right)+\left(0.0044\cdot \mathrm{IL}\text{-}18\left[\frac{\mathrm{pg}}{\mathrm{mL}}\right]\right)\\ +\left(2.6159\cdot \mathrm{phosphates}\left[\frac{\mathrm{mg}}{\mathrm{dL}}\right]\right)+\left(-8.8762\cdot \mathrm{ALB}\left[\frac{g}{\mathrm{dL}}\right]\right) \end{array}$$

The positive value of the model’s result denoted with the letter *Y* is associated with a high risk of death, while values below zero correspond to decreased risk. The model was able to correctly assign 95.5% of the patients in the HD group to HD-D and HD-A accordingly. The model mean accuracy was 0.8893 ± 0.0617, and the random sampling of the data estimated the train (80%) and test (20%) sets; cross-validation was repeated 20 times. General information about the model can be found in Table 4.

An evaluation of the chosen predictor variables has been summarized in Table 5, in which the Wald test results are presented. All variables seem crucial for the model (relatively high z value). Although the p value for 3-NT is above the typical threshold (*p* > 0.05), deletion of this variable worsens the model.

**Figure 6:**
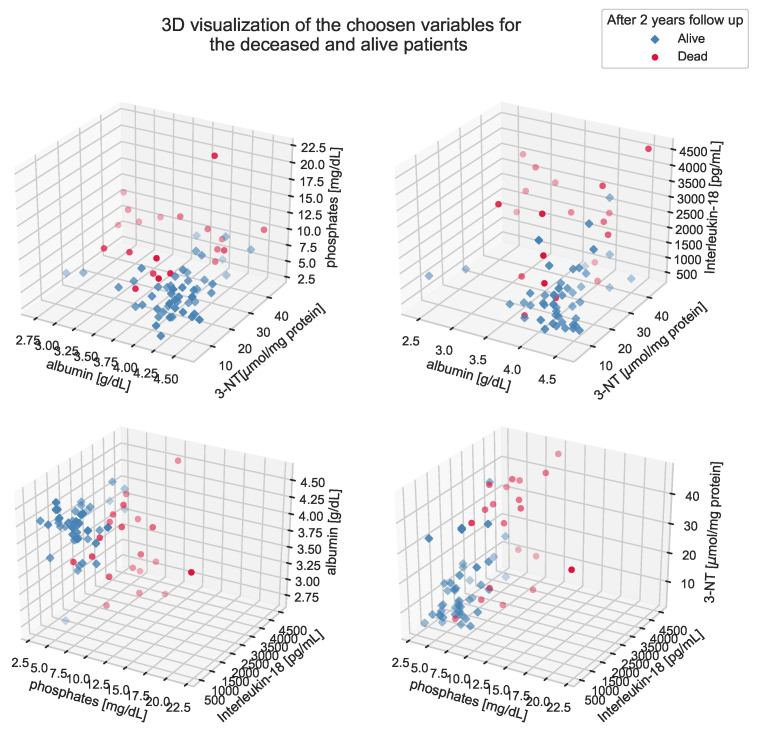
Three-dimensional visualization of spacious relationships between the predictor variables. The HD-A and HD-D groups are marked with blue and red colors, respectively. For the sole purpose of creating a better perception of distances between positions, a color transparency gradient, which highlights depth of the images, has been added.

**Figure 7:**
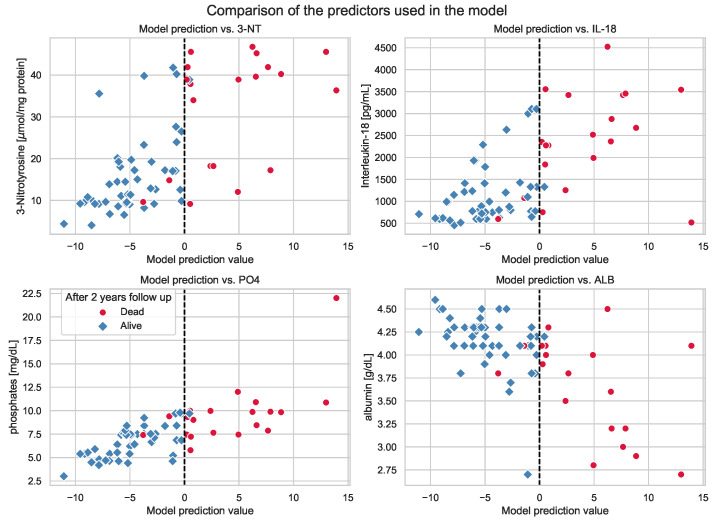
Visualization of the model results with respect to all the four chosen predictors. The HD-A and HD-D groups are marked with blue and red colors, respectively. The vertical dashed line represents a change in the predicted fate of patients (the negative value of the model prediction can be treated as predicting that a patient will survive in the next 2 years, but a positive value foreshadows the patient’s death).

According to Figure 6, it can be assumed that a multidimensional structure of relationships between the predictors allows for potently good separation of the HD-A and HD-D groups. Based on the visualization of the model results showing the relationship between the prediction and a single predictor value (Figure 7), it is possible to see that sole variables are not sufficient to provide a good model. Therefore, a combination of four chosen variables together, in our opinion, is able to predict patient death with high accuracy.

**Figure 8:**
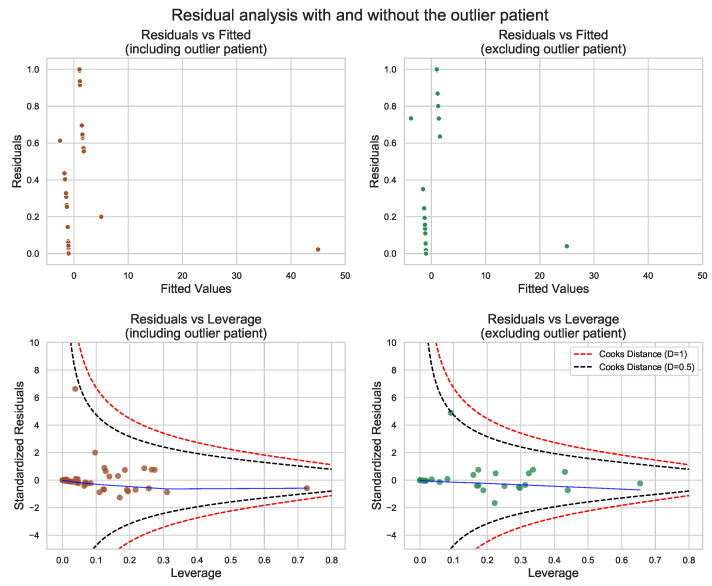
Residual diagnostic plots for the models with and without the outlier patient. Outliers can be spotted as outfits in residual vs. fitted plots. The significance of outliers can be analyzed by residuals vs. leverage plots. Sole observations at the end of the blue line may have a significant impact on the model if they cross the dashed lines drawn for Cook’s distance. In the presented case, the outlier has not fallen outside the Cook’s boundaries, and as it is presented in the subplots on the right, its deletion does not change the model in any significant manner.

During residual analysis, a strong outlier patient was detected. Based on the obtained results, it can be concluded that the deletion of this outlier will not have any significant impact on the final model. In order to double check the model, the outlier patient was deleted from the dataset and a residual analysis was performed; results are presented in Figure 8. The obtained results clearly indicate that the differences between the models with and without the outlier patient are negligible.

The statistical approach that helped to obtain the model is limited by the number of dead patients. Although the detected relationships seem to be clear and strong, it is reasonable to expect some sampling bias. It is reasonable to suppose that this model could be easily adopted in clinical practice and used to prevent patient death, if possible.

## 4. Discussion

The pathogenesis of CKD-related CVD is complex and multifactorial, primarily as renal dysfunction is caused by various disturbances, including autoimmune, metabolic, oncological, and vascular diseases.

Today, there is no doubt that the diagnosis of CKD poses an increased risk of CVD [1]. Although we already know a lot about the causes of civilization diseases, research is still ongoing to better understand the mechanisms underlying cardiovascular disorders in CKD. This is important because CVD is the greatest challenge in this group of patients. Commonly recognized traditional risk factors that are known to be significant in patients without kidney damage appear to be significantly less important in CKD patients, and the more severe the kidney impairment, the less their significance appears to be.

The results of our research are another voice indicating that in CKD, looking for the causes of increased CV-related morbidity and mortality solely in the group of traditional factors commonly considered as CV will only be in vain. The present study shows that the causes of CV-related mortality in HD patients are complex and should be considered in the context of the contribution of not one singe factor, but of the entire system of relationships between various factors. This mixture of dependencies is quite a challenge.

Based on the results of the statistical analysis, we discovered that the four parameters—IL-18, 3-NT, phosphate, and ALB—when taken together in one proposed model, could predict 95.5% of the CV-related death over a 2-year follow-up among the hemodialyzed patients included in this study. These four parameters represented the main problems of HD patients: inflammation, oxidative stress, malnutrition, and calcium-phosphate disorders.

### 4.1. Inflammation-IL-18

Among the unique features of CKD patients, chronic inflammation is one of the key issues. On the one hand, the inflammatory process is the body’s protective response to infection and tissue damage, which leads to vasodilatation and the recruitment of leukocytes, plasma proteins, and fluid into the affected tissue. On the other hand, chronic inflammation may be a maladaptive and poorly controlled response (e.g., in uremia) that contributes to many complications, including cardiovascular ones. It is worth noting that chronic inflammation occurs in a large proportion of the CKD population and is increasingly associated with renal function deterioration [5].

The inflammasome—an intracellular platform created in response to pathogen-associated molecular patterns (PAMP) and danger-related molecular patterns (DAMP)—by converting procaspase-1 into active caspase-1, activates pro-inflammatory cytokines such as interleukin 1-β (IL-1β) and IL-18, thus triggering cell pyroptosis [19,20]. Recent studies have focused on the nucleotide-binding domain, leucine-rich repeats, pyrine-containing domain 3 (NLRP3) inflammasome role, which is widely activated in sterile inflammation [20,21]. NLRP3 is a sensor of cellular stress induced by both various external (pathogens) and internal factors (metabolic imbalance, organelle damage, and mitochondrial oxidative stress). During kidney injury, DAMPs such as ATP, uric acid, mitochondrial ROS, and extracellular matrix components are released and activate the NLRP3 inflammasome [22]. As discovered in several studies, this activation can contribute to interstitial fibrosis and CKD. Liu et al. [23], in a model of nephropathy in rats, revealed that albuminuria may contribute to tubulointerstitial inflammation in CKD via mitochondrial ROS-mediated inflammasome activation. Moreover, NLRP3-augmented intrarenal expression among non-diabetic patients with kidney disease has been found to be associated with worse renal function, suggesting that the NLRP3 inflammasome contributes to CKD [24]. The action of the NLRP3 inflammasome-activating IL-18 is related to the leakage of ROS from the mitochondria, suggesting an association between IL-18 and oxidative stress. ROS can also detach the ROS/thioredoxin-interacting protein (TXNIP) from the endogenous antioxidant thioredoxin, which allows TXNIP to react with the NLRP3 and activate the cascade, leading to the synthesis of pro-inflammatory cytokines [25].

In our proposed predictive CV-mortality model, the inflammatory process was represented by IL-18, a cytokine that can regulate inflammation at multiple checkpoints [20]. Multifunctionality and participation in inflammatory cascades as well as innate and acquired immunity makes IL-18 an essential immune actor. High serum levels of IL-18 have recently been identified as a strong predictor of death in patients with coronary artery disease and acute ischemic stroke [26]. The principal mechanism of IL-18-mediated cardiovascular events is based on its ability to induce IFN-γ synthesis in T cells and natural killers. IFN-γ contributes to the thinning of the fibrous cap of the atherosclerotic plaque by stimulating the expression of adhesion molecules on the endothelium, and the complex II on macrophages and vascular cells. It has been found to promote the inhibition of collagen synthesis in cells, increasing the expression of matrix metalloproteinases [27]. As the adaptive immune system is involved in the development of atherosclerosis, the importance of IL-18 is considered crucial, especially when we focus on CKD-related atherosclerosis and its clinically relevant consequences [28]. Although data on the role of IL-18 in predicting coronary events are contradictory [29,30], many studies have shown its utility as a strong independent predictor of death from CV causes in patients with coronary artery disease regardless of the clinical status at admission [26,31,32]. This strongly supports the evidence of an IL-18-mediated inflammatory process, leading to the acceleration and vulnerability of the atherosclerotic plaque. Moreover, it has been disclosed that the inflammasome-dependent activation of IL-18 within the myocardium upon the acute activation of the β-adrenergic receptor has triggered cytokine cascades, macrophages infiltration, and pathological cardiac remodeling [32].

In the previous study by Formanowicz et al. [33], in a 2-year follow-up, it was found for the first time that serum IL-18 concentration might reflect the risk of CV death among HD patients with AMI in the previous year. However, the study focused mainly on IL-18 and did not consider as many different parameters as in the present study. Nevertheless, as in the present study, the HD patients in the previous study with high serum levels of IL-18 experienced worse survival than patients with lower IL-18 levels.

In this study, both HD groups have shown significantly higher levels of IL-18 in the serum than in the control group. There are at least several possible explanations for this condition. First, IL-18 is a medium molecule and protein-bound uremic toxin that is difficult to remove with any of the current dialysis strategies; hence, the observed accumulation of IL-18 in dialysis patients. Second, the dialysis procedure activates the monocyte/macrophage network and synthesizes many inflammatory cytokines, explaining the observed increase in serum IL-18. Moreover, IL-18 has been associated with the instability of the atherosclerotic plaque, which might have also increased its concentration in the studied patients. Additionally, nitric oxide (NO) has been revealed to suppress the secretion of IL-1β and IL-18 by inhibiting caspase-1 [19]. Hence, the NO changes in the course of oxidative/nitrosative stress may contribute to an increase in IL-18 synthesis in CKD.

Our previous results [34] confirmed that IL-18 and IFN-γ play a key role in atherosclerosis. In addition, we showed that the impact of renal failure significantly accelerates this pathology. Moreover, we proved that IL-18 along with the directly dependent signaling pathways (IL-18-MyD88 axis) are crucial for atherosclerosis and can be considered as a good candidate for an atherosclerosis biomarker. Comparing the different variables of a widely recognized impact on CV risk, we showed the advantage of IL-18 over other parameters such as IMT and albumin (moderately predictive), as well as NT-proBNP, hsCRP, eGFR, and albumin (less predictive). Moreover, IL-18 also proved to be the best predictor for a two-year CV mortality in the stepwise regression [33,35]. In this study, we confirmed the IL-18 significance in CV-related mortality among HD patients. On the other hand, the well-established CV biomarker hsCRP was not included in the predictive model because it did not allow for a differentiation between HD-A and HD-D, although the serum concentration of hsCRP was increased in both HD groups as compared to the control. This may be the result of having a relatively small study group, as well as the overlapping participation of many factors.

### 4.2. Oxidative Stress/Nitrosative Stress-3NT

One of the most significant causes of CVD in CKD is increased oxidative stress and the insufficient effectiveness of antioxidant systems. The redox imbalance negatively affects all components of the kidneys, i.e., the renal circulatory system, glomeruli, renal tubules, and interstitial tissue. Furthermore, HD, the method of renal replacement therapy, bothers the redox balance. An additional source of oxidative stress is the treatment—heparin use, which increases the activity of MPO. The issues of oxidative stress in the context of kidney disease and the effects on the cardiovascular system are discussed in detail in [5].

In addition to the significance of oxidative stress, the role of nitrosative stress, characterized by the overproduction of NO, has also been emphasized [36]. It is responsible for endothelial dysfunction caused by the decoupling of nitric oxide synthase (NOS), oxidative damage to lipids, proteins, and DNA in vascular endothelial cells related to inflammation and atherosclerosis. Hence, it has been found that oxidative and nitrosative stress play an important role in increasing arterial stiffness in many diseases, including diabetes mellitus, hypertension, obesity, peripheral arterial disease, and metabolic syndrome [37]. Cardiovascular risk factors reduce endothelial NO production and stimulate ROS production from a variety of sources, including NADPH oxidase, xanthine oxidase (XO), mitochondria, and uncoupled eNOS. ROS and NO play opposite roles in the process of atherosclerosis, such as LDL oxidation, endothelial cell activation, and macrophage infiltration/activation.

Reactive nitrogen species (RNS), similar to ROS, are characterized by a significant oxidizing potential and a short half-life, resulting from their ability to react with other molecules. One of the products of RNS action on proteins is 3-NT, which is most often formed by the indirect modification of the tyrosine residue. At the first stage, the attack of ONOO${}^{-}$ on the tyrosine residue results in the synthesis of the unstable tyrosyl radical, which is then converted to 3-NT. An alternative pathway involves dimerizing the tyrosyl radicals, leading to the appearance of 3,3-dityrosine and cross-links within the protein molecule [38]. Enzymes from the peroxidase group are significantly involved in synthesizing 3-NT: myeloperoxidase (MPO) and eosinophilic peroxidase, produced by inflammatory leukocytes in the course of hypersensitivity reactions. Studies with animal models have revealed significant differences in the participation of peroxidases in the formation of 3-NT, depending on the location and inflammation etiology. Moreover, the intensification of the synthesis of 3-NT does not correlate with the intensity of the formation of other products of peroxidase activity. Due to the possibility of a limited influx of nitrite (NO${}^{2-}$) in vivo, peroxidases play a more significant role in the nitration of amino acid residues in extracellular proteins than inside the cell [26].

In the present study, the accumulation of 3-NT has been found in both HD groups when compared to control, and a significant difference between HD-A and HD-D has been disclosed. However, different results were obtained by Massy et al. [39], who evaluated the concentration of nitrated and nitrosylated derivatives in HD patients. While the concentration of S-nitrosothiols was significantly higher in the HD group, there was no difference in the concentration of 3-NT between the HD group and the healthy group. It should be noted that this study involved a small group of only 22 HD patients [39]. In turn, Kose et al. assessed the concentration of 3-NT in patients treated with various renal replacement techniques. Only patients in the HD group were found to have elevated levels of 3-NT, both before and after HD. In contrast, in peritoneal dialysis patients, the concentration of 3-NT did not differ from that in healthy volunteers [40]. The other studies conducted by Piroddi et al. [41], Namiduru et al. [42], and Mitrogianni et al. [43], based only on HD patients, revealed significantly elevated 3-NT concentration in the HD vs. control groups. Moreover, no differences were found in the 3-NT concentration before and after dialysis, regardless of the type of dialyzer that was used. The dominance of the group of HD patients has raised the question of the impact of renal replacement therapy on the development of nitrogen stress [43]. It is worth noting that one of the possible routes for the formation of 3-NT is the synthesis by MPO, the key enzyme in the oxygen burst. The CKD impact on MPO activity and serum concentration has not been clearly established; some of the studies have indicated a negative correlation between MPO and urea and creatinine, or the unchanged serum concentration of MPO. On the contrary, in other studies on patients treated with renal replacement therapy, an increase in the concentration and activity of MPO was reported [44]. Namiduru et al. revealed elevated 3-NT and MPO in HD [42].

Kidney damage has also been shown to be associated with nitrosative stress in animal models. In rats, after 5/6 kidney nephrectomies, the accumulation of 3-NT has been observed in the rest of the kidney tissue as well as in other organs (heart, aorta, brain, liver). At the same time, a decrease in the concentration of iNOS and endothelial NOS (eNOS) in the kidneys, heart, and aorta has been observed, suggesting disturbances in the synthesis of NO, resulting in vasoconstriction [45]. Cardiovascular risk factors have contributed to the decrease in endothelial NO production, and they have stimulated ROS generation from various ROS sources, including NOXs, XO, mitochondria, and uncoupled eNOS. ROS and NO have shown the opposite roles in the process of atherogenesis, such as LDL oxidation, endothelial cell activation, and macrophages infiltration/activation.

Recent research findings have suggested the existence of a potential link between the formation of NO-derived oxidants and the development of CAD [46,47]. It has been underlined that the presence of an imbalance between O${}_{2}^{•-}$ and NO formation within diseased arterial walls leads to a functional NO deficiency and consequently, the production of NO-derived oxidants. Human monocytes have been shown to use numerous (ONOO${}^{-}$ and MPO)-dependent pathways to produce NO-derived oxidants, and 3NT is one of them. One consequence of these reactions is the oxidative conversion of LDL. Other mechanisms have linked NO-derived oxidants to the activation of matrix metalloproteases and the development of unstable atherosclerotic plaques and pro-thrombotic states. Although the increase of 3-NT in human atherosclerotic lesions is already well-understood in both immunohistochemical and mass spectrometric studies, Shishehbor et al. [46] were the first researchers to have shown that systemic 3-NT levels directly correlate with the presence of CAD and can respond to statin therapy. These investigators have shown an interesting potential clinical utility in using nitrotyrosine levels as an adjunct marker in the stratification of CAD risk and the monitoring of the anti-inflammatory effects of statin therapy.

In the current study, for the first time, we discovered that 3-NT, the representative molecule of nitrosative stress, could be a promising marker of CV mortality.

### 4.3. Malnutrition-Inflammation-Albumin

Malnutrition is one of the most serious CKD complications. The global incidence of malnutrition in kidney disease is 28–54%, [48] and it is associated with a higher risk of death, ranging from 1.61 to 4.08 [49,50]. There are different definitions for malnutrition, including protein-energy wasting (PEW), protein-energy malnutrition, malnutrition–inflammation complex syndrome, malnutrition–inflammation–atherosclerosis (MIA syndrome), and uremic wasting syndrome, depending on the involvement of inflammation, hypercatabolism, and increased uremia [51,52].

Malnutrition in CKD is associated with an increased incidence of CVD and the rapid progression of atherosclerotic organ damage. Moreover, it has been proven that along with the deterioration of kidney function, there is an enhanced secretion of pro-inflammatory cytokines, resulting in increased resting energy consumption (a link between malnutrition and oxidative stress has been suggested to be related to the NLRP3 inflammasome). This leads to the intensification of catabolism and malnutrition [53].

In general, the causes of malnutrition in HD patients are varied and include numerous factors that interact with each other, among which the following are highlighted: (1) reduced energy or protein intake; (2) coexistence of chronic diseases and overlapping acute diseases; (3) the HD procedure itself acting as a catabolic stimulus; (4) possible loss of water-soluble vitamins and nutrients into the dialysate during HD; (5) the significantly altered intestinal microbial flora; (6) diagnostic or therapeutic procedures that can reduce nutrient intake or cause net protein breakdown; (7) chronic blood loss; (8) hormonal derangements with particular emphasis on insulin resistance and an impaired balance between pro-inflammatory leptin and anti-inflammatory adiponectin; (9) metabolic products that accumulate in renal failure and may contribute to wasting; (10) loss of renal metabolic function; and (11) accumulation of toxic substances taken from the environment. All of these factors taken together may promote oxidative, nitrosative, and carbonyl stress, anemia, acidosis, and chronic inflammatory processes [5].

The relationship between serum albumin concentration and increased mortality among HD patients was first discovered by Goldwasser [54]; numerous studies have subsequently confirmed it. It has been revealed that a serum albumin level of less than 3.8 g/dL (and/or a reduction in serum albumin levels) confers a greater mortality risk in patients with kidney failure [55]. In addition, several observational studies have shown an association of low albumin concentration with poor treatment outcomes in HD patients, where the odds ratio of mortality increased along with the decrease in serum albumin level; serum albumin was (after the age of the studied patients) the strongest predictor of mortality [56,57]. However, Mukai et al. [58] found that although low serum albumin is common in dialyzed patients and is associated with poor prognosis, it may be more of an indicator of persistent inflammation than an indicator of nutritional status. Additionally, Mutsert et al. [59] concluded that the relationship between serum albumin levels and mortality could be explained in part by inflammation rather than by nutrition. Moreover, Thijssen et al. [60] noted that serum albumin levels may be affected by interactions between inflammation, nutrition, and dialysis efficiency.

In this study, both HD groups showed significantly lower serum albumin levels than the control group. Moreover, HD-D patients disclosed a significantly lower serum albumin concentration than the HD-A group. The median serum albumin concentration in HD-A was 3.85 g/dL, which, according to the results of various studies on HD patients, made the albumin level a significant biomarker of increased mortality in this group. It should be mentioned that no assessments have been performed to check the nutritional status of the patients studied; only BMI was calculated. BMI values revealed that in the HD-D group, more people were overweight than in HD-A. The observed hypoalbuminemia could be a result of the influence of many factors and inflammation in both groups, since albumin acts as a negative acute-phase reactant and impacts the nutrition status in some particular studied patients.

### 4.4. Calcium-Phosphate Disturbances-PO${}_{4}^{3-}$

Phosphate homeostasis in the human organism is directly influenced by calcitriol, PTH, fibroblast growth factor 23 (FGF-23), and Klotho protein. CKD is considered as a Klotho-deficiency state and a premature aging disease [61]. The kidney plays a key role in this homeostasis since as much as 90% of the daily phosphate load is excreted by this organ. When the disease begins to affect renal function, there is a compensatory enlargement of the remaining nephrons, and an increased rate of filtration to nephrons is observed. To maintain homeostasis, compensating for the progressive loss of the nephron’s working mass, the excretion of phosphate into the nephron is also increased. Increased levels of PTH and FGF-23 mediate increased phosphate excretion per functioning nephron in early stage renal disease. In this way, it is initially possible to maintain the correct level of phosphate in the plasma. As kidney function gradually declines, increasing levels of PTH are needed to maintain phosphate homeostasis. In advanced stages of kidney disease, when the excretory function of the kidneys is significantly reduced, elevated levels of PTH, which can act as a uremic toxin in kidney failure, cannot maintain normal phosphate levels, and hyperphosphatemia becomes evident. Renal failure also leads to decreased calcitriol synthesis and secondary hyperparathyroidism, resulting in increased bone reabsorption and the release of calcium and phosphate into the circulation. The frequently observed metabolic acidosis in renal failure may also contribute to hyperphosphatemia by shifting phosphate out of the cells.

Hyperphosphatemia, due to its contribution in hypertension, vascular calcification, calcification of heart valves, atherosclerosis, left ventricular hypertrophy, and myocardial fibrosis [62], has been found to be a significant risk factor for CV mortality among CKD patients [63,64]. It has been shown in numerous studies that even among patients with moderate renal impairment, higher serum phosphate levels were associated with vascular calcification [62,65,66,67,68]. Overall, the clinical and experimental data have emphasized that high phosphate levels may contribute to vascular calcification in CKD patients, but are also crucial in the general population.

In our current study, significantly higher phosphate levels have been observed in HD patients compared to the control group. Additionally, serum phosphates differentiated the HD-D and HD-A groups, which confirms previous reports on the importance of hyperphosphatemia. These results clearly show that special emphasis should be placed on reducing hyperphosphatemia as it is by far one of the most important modifiable mortality factors in CKD.

### 4.5. Other Findings Disclosed Based on Comparisons between CKD-A and CKD-D

In addition, apart from the previously presented markers that were selected for the predictive model, the most significant differences between the HD-A and HD-D groups were found for the NT-proBNP and AOPP serum concentration. In this study, both HD groups showed significantly higher levels of Nt-proBNP compared to healthy people, which is not surprising because people who survived AMI in the previous year were included in the study. It should be emphasized that the HD-D patients revealed the highest concentrations of Nt-proBNP, which proves their poor cardio-vascular system condition.

On the other hand, AOPP, known biomarkers of the oxidative proteins modification [69], also turned out to be significantly different in the HD-A and HD-D groups. Additionally, the concentration of AOPP in serum increased with the increase in 3-NT; however, 3-NT and not AOPP proved to be a better predictor of the 2-year cardiovascular mortality in HD patients. The observed importance of AOPP in discriminating between the HD-A and HD-A groups is in line with the recent findings by Zhou et al. [70]. These researchers revealed that elevated serum AOPP levels were associated with a higher risk of all-cause mortality in 1394 Chinese HD patients, during a median follow-up duration of 5.2 years. These results are essential because there is a lack of long-term studies on this topic, and the previous small sample-based studies produced conflicting results [71].

Interestingly, the 2-year CV mortality predictive model that has been discovered in this study does not contain parameters directly indicative of renal dysfunction, such as urea, creatinine, or eGFR. Surprisingly, the urea concentration was even lower in HD-D vs. HD-A. Furthermore, changes in the lipid metabolism parameters did not explain the increased mortality in the HD-D group. In addition, the results obtained from non-invasive cardiac examinations proved to be insufficient predictive support. However, there were some differences between HD-A and HD-D, mainly in the central rather than the peripheral blood pressure (pESP, cESP, cAP, and cMPD). HD-D patients, when compared to HD-A, revealed a thicker atherosclerotic plaque (based on the increased IMT values), which were mostly more unstable (based on hsCRP values). It should be emphasized that both HD groups had a significantly larger atherosclerostic plaque than the control group, and in both HD groups, hsCRP in serum was significantly higher than in the control group.

Moreover, in the study by Wang et al., a positive correlation between hsCRP and the area of the coronary plaque was found, suggesting an important relationship between hsCRP and the local inflammatory process [72]. It could explain the observed increase in hsCRP serum concentrations in both studied HD groups involving patients after AMI in the previous year. Additionally, it should be pointed out that the HD-D patients were significantly older than those of the HD-A group. According to our previous research results, age might also affect hsCRP serum concentration, which should also be considered here [73,74]. In fact, age was not found to significantly affect the mortality rate among studied HD patients and has not been discovered to be a good candidate as a CV mortality predictor in a 2-year follow-up.

In summary, inflammation, oxidative stress/nitrosative stress, malnutrition, and calcium-phosphate imbalance are closely related processes seen in HD patients. In addition to the hypotheses put forward in the mid-1980s, which linked the exposure of whole blood to cellulose membranes with increased IL-1 synthesis as the cause of HD disorders, it was not until the late 1990s that the first reports of uremia and its association with CVD, PEW, and worse survival outcomes appeared. What at the time was called the “new” risk factor has now evolved into an established, non-traditional risk factor. The results that have been obtained in this study may indicate an advantage of non-traditional risk factors over traditional risk factors in predicting CV-related mortality in HD patients. Not all parameters differentiating the HD group were finally included in the predictive model that we proposed; however, the addition of the rest, namely AOPP and NT-proBNP, has not changed the predictive power of the proposed model, which does not exclude their importance.

## 5. Conclusions

It has been discovered that four interrelated processes, such as immuno-inflammatory processes, nitrosative and oxidative stress, malnutrition, and calcium-phosphate disorders, may be essential, especially if they are taken into account together, in predicting CV-related mortality in HD patients with a history of AMI in the previous year prior to enrollment in the study.

Moreover, the results of our studies have shown that it is most likely impossible to use only one marker to predict cardiovascular risk in hemodialyzed patients, mainly due to the complexity of the mechanisms underlying the increased cardiovascular risk in this group. Understanding the interplay between kidney and heart is still quite a challenge, and routinely used markers, known as traditional markers, are most often not very efficient at predicting mortality in this remarkable group of patients.

## Figures and Tables

**Table 1:** Basic information about the groups (MEAN ± SD or % in the case of Sex, Smoking, and Overweight). Statistical significance given in respective columns was calculated based on the $\chi^{2}$ test for data on Sex, Smoking, and Overweight. In the remaining cases, the *p*-value was taken from the results of the Kruskal–Wallis H test. Since the group HD-D in contrast to HD-A and Control contains only patients who died, there was no point in calculating the *p*-value for survival from study entry. The comparison of the duration of HD treatment took into account only two groups (HD-A and HD-D). Significance of differences found is marked in following way: ***—p≤0.001; **—0.001<p≤0.01; *—0.01<p≤ 0.05; X—no significant difference.

	*p*-Value	HD-D (n=20)	HD-A (n=51)	Control (n=32)
Age (years)	*	64.85 ± 11.09	54.63 ± 15.22	60.28 ± 12.49
Sex (% female)	*	0.30	0.33	0.47
Smoking (%)	***	15.00	23.50	6.25
BMI (kg/m${}^{2}$)	**	24.45 ± 3.77	22.37 ± 3.50	25.04 ± 4.05
Overweight (%)	***	40.00	13.70	34.38
Urea (mg/dL)	***	88.45 ± 41.18	109.83 ± 41.22	27.79 ± 8.77
Creatinin (mg/dL)	***	6.82 ± 2.83	8.4 ± 2.54	0.62 ± 0.11
eGFR (mL/1.73 m${}^{2}$/min)	***	8.98 ± 7.02	5.94 ± 3.80	111.75 ± 24.07
hsCRP (mg/L)	***	14.11 ± 10.56	9.55 ± 4.49	2.04 ± 1.44
Duration of HD				
treatment (months)	X	19.7 ± 13.67	25 ± 5.61	–
Survival from				
study entry (months)	–	11.85 ± 4.77	>24	>24

**Table 2:** Significant differences for HD-A vs. HD-D (calculated Kruskal–Wallis $H>H_{critical}\;\left(5.991\right)$ on proper level of significance p≤0.05), with additional comparison to existing (or not) dissimilarities to the control group (HD-D vs. Control and HD-A vs. Control).

Variable	HD-D vs. HD-A		HD-D vs. Control		HD-A vs. Control	
	*H*	*p*-Value	*H*	*p*-Value	*H*	*p*-Value
Age	6.6758	0.0098	3.7989	0.0513	2.9363	0.0866
3-NT	16.2729	0.0001	36.2356	<$10^{-4}$	54.0889	<$10^{-4}$
IL-18	14.5679	0.0001	36.2388	<$10^{-4}$	56.9062	<$10^{-4}$
ALB	13.1005	0.0003	21.1171	<$10^{-4}$	6.8533	0.0088
ALP	10.5967	0.0011	13.7319	0.0002	0.2846	0.5937
AOPP	11.3928	0.0007	36.2326	<$10^{-4}$	57.7278	<$10^{-4}$
NT-proBNP	10.9114	0.0009	35.3365	<$10^{-4}$	52.8080	<$10^{-4}$
PO${}_{4}^{3-}$	19.673	<$10^{-4}$	36.2775	<$10^{-4}$	53.2101	<$10^{-4}$
IMT	7.8134	0.0051	15.5445	0.0001	4.8827	0.0271
pSP	6.5922	0.0102	22.4169	<$10^{-4}$	9.0008	0.0027
pMEANP	6.3964	0.0114	23.4074	<$10^{-4}$	13.9504	0.0002
pP2	9.4061	0.0022	23.3944	<$10^{-4}$	11.4182	0.0007
pESP	7.4748	0.0063	16.5906	<$10^{-4}$	3.39187	0.0655
cP2	9.9641	0.0016	22.3157	<$10^{-4}$	8.7491	0.0031
cESP	9.1720	0.0024	17.9297	<$10^{-4}$	4.8384	0.0278
cAP	7.1287	0.0076	8.39942	0.0038	0.9295	0.335
cMPS	8.3386	0.0038	22.8494	<$10^{-4}$	10.8557	0.001
cMPD	6.3318	0.0119	15.9177	0.0001	4.1257	0.0422
cPH	10.1264	0.0014	19.8839	<$10^{-4}$	10.2415	0.0014
cSP	8.10444	0.0044	20.2442	<$10^{-4}$	15.0661	0.0001
pPP	9.73906	0.0018	22.6132	<$10^{-4}$	15.7465	0.0001

**Table 3:** Significant differences for HD-A vs. HD-D (calculated Kruskal–Wallis $H>H_{critical}\;\left(5.991\right)$), with additional comparison to existing (or not) dissimilarities to the control group (HD-D vs. Control and HD-A vs. Control). Significance of differences found has been marked in following way: ***—p≤0.001; **—0.001<p≤0.01; *—0.01<p≤ 0.05; X—no significant difference.

Variable	HD-D vs. HD-A	HD-D vs. Control	HD-A vs. Control
Age	**	X	X
3-NT	***	***	***
IL-18	***	***	***
ALB	***	***	**
ALP	**	***	X
AOPP	***	***	***
NT-proBNP	***	***	***
PO${}_{4}^{3-}$	***	***	***
IMT	**	***	X
pSP	*	***	**
pMEANP	**	***	***
pP2	**	***	***
pESP	**	***	X
cP2	**	***	**
cESP	**	***	X
cAP	**	**	X
cMPS	**	***	***
cMPD	*	***	X
cPH	**	***	**
cSP	**	***	***
pPP	**	***	***

**Table 4:** Detailed information about the model separating the HD-D and HD-A groups.

Parameter	Value
Model family	Binomial
No. observations	67
Residual degrees of freedom	62
Model degrees of freedom	4
Link function	Logit
Method	IRLS
Scale	1.000
Log-likelihood	−13.749
Deviance	27.498
Pearson chi2	58.8
No. iterations	8
Covariance type	nonrobust

**Table 5:** The Wald test results for the chosen combination of predictor variables (3-NT, IL-18, PO${}_{4}^{3-}$, and Albumin). The more that the z value is away from 0, the more likely it is that excluding the analyzed variable will significantly harm the model. The 0.025 and 0.975 columns show boundaries for a 95% confidence interval for the coefficients.

Predictor	Coefficient	Standard Error	z	*p*-Value > |z|	0.025	0.975
Intercept	1.5255	5.769	0.264	0.791	−9.781	12.832
3-NT	0.057	0.04	1.451	0.147	−0.02	0.136
IL-18	0.002	0.001	2.510	0.012	0.0	0.004
PO${}_{4}^{3-}$	1.2045	0.474	2.541	0.011	0.275	2.134
Albumin	−4.2076	2.026	−2.077	0.038	−8.178	−0.237

## Data Availability

All necessary data are included in the paper.

## Supplementary Materials

The following are available at https://www.mdpi.com/article/10.3390/antiox11020355/s1, Figure S1: Spearman correlation results heat map for hemodialyzed patients and control group, Table S1: Spearman correlation test results for all variables that significantly differentiate groups HD-A and HD-D.
