# Supplementary material for: The Mutual Contribution of 3-NT, IL-18, Albumin, and Phosphate Foreshadows Death of Hemodialyzed Patients in a 2-Year Follow-Up
Source: Antioxidants (Basel). 2022 Feb 11;11(2):355. doi: 10.3390/antiox11020355 (PMC8868576; doi:10.3390/antiox11020355)
Supplement: Supplementary file 1 [file antioxidants-11-00355-s001.zip › Figure S1 and Table S1.pdf]

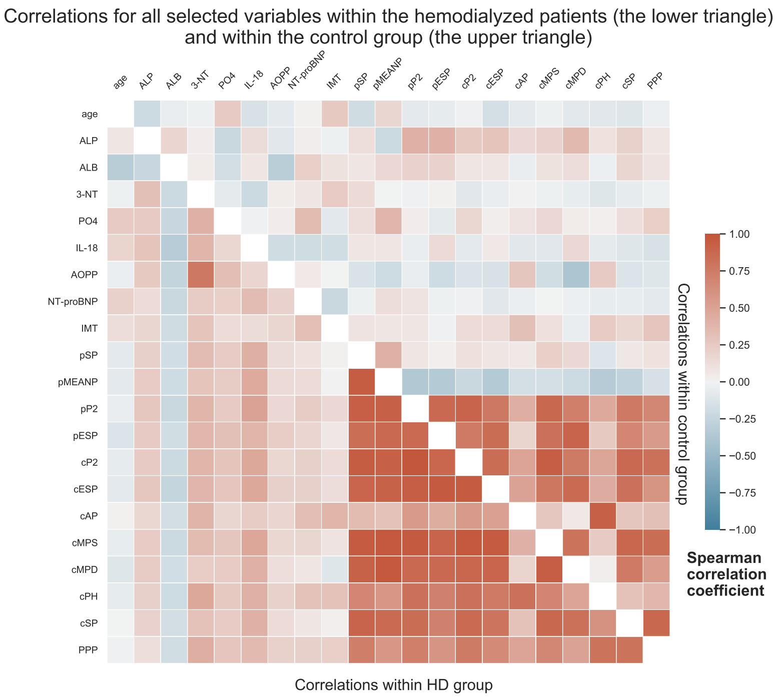

Figure S1: Spearman correlation results heat map for hemodialyzed patients (lower triangle) and control group (upper triangle). Positive correlations ($\rho > 0$) are marked with red color and negative ($\rho < 0$) are presented as blue. Color intensity reflects of correlation coefficient (ρ) (the more intense color is, the more away from 0 is the correlation coefficient).

Table S1: Spearman correlation test results for all variables that significantly differentiate groups HD-A and HD-D.

**Variable 1**	**Variable 2**	**Spearman ρ**	**p-value**
3-NT	AOPP	0.7738	$< 10^{-4}$
IL-18	pP2	0.5015	$< 10^{-4}$
pSP	pMEANP	0.9463	$< 10^{-4}$
pSP	pP2	0.9345	$< 10^{-4}$
pSP	pESP	0.8361	$< 10^{-4}$
pSP	cP2	0.9517	$< 10^{-4}$
pSP	cESP	0.8976	$< 10^{-4}$
pSP	cMPS	0.9601	$< 10^{-4}$
pSP	cMPD	0.9183	$< 10^{-4}$
pSP	cPH	0.6832	$< 10^{-4}$
pSP	cSP	0.9042	$< 10^{-4}$
pSP	PPP	0.7365	$< 10^{-4}$
pMEANP	pP2	0.9250	$< 10^{-4}$
pMEANP	pESP	0.8705	$< 10^{-4}$
pMEANP	cP2	0.9270	$< 10^{-4}$
pMEANP	cESP	0.9194	$< 10^{-4}$
pMEANP	cMPS	0.9759	$< 10^{-4}$
pMEANP	cMPD	0.9737	$< 10^{-4}$
pMEANP	cPH	0.5821	$< 10^{-4}$
pMEANP	cSP	0.8549	$< 10^{-4}$
pMEANP	PPP	0.5794	$< 10^{-4}$
pP2	pESP	0.8646	$< 10^{-4}$
pP2	cP2	0.9889	$< 10^{-4}$
pP2	cESP	0.9424	$< 10^{-4}$
pP2	cAP	0.5617	$< 10^{-4}$
pP2	cMPS	0.9613	$< 10^{-4}$
pP2	cMPD	0.8688	$< 10^{-4}$
pP2	cPH	0.8139	$< 10^{-4}$
pP2	cSP	0.8440	$< 10^{-4}$
pP2	PPP	0.7314	$< 10^{-4}$
pESP	cP2	0.8896	$< 10^{-4}$
pESP	cESP	0.9676	$< 10^{-4}$
pESP	cMPS	0.9013	$< 10^{-4}$
pESP	cMPD	0.8745	$< 10^{-4}$
pESP	cPH	0.7126	$< 10^{-4}$
pESP	cSP	0.7149	$< 10^{-4}$
pESP	PPP	0.5937	$< 10^{-4}$
cP2	cESP	0.9558	$< 10^{-4}$
cP2	cAP	0.5486	$< 10^{-4}$
cP2	cMPS	0.9718	$< 10^{-4}$
cP2	cMPD	0.8839	$< 10^{-4}$
cP2	cPH	0.8174	$< 10^{-4}$
cP2	cSP	0.8596	$< 10^{-4}$
cP2	PPP	0.7559	$< 10^{-4}$
cESP	cAP	0.5143	$< 10^{-4}$
cESP	cMPS	0.9589	$< 10^{-4}$
cESP	cMPD	0.8961	$< 10^{-4}$
cESP	cPH	0.7613	$< 10^{-4}$
cESP	cSP	0.8015	$< 10^{-4}$
cESP	PPP	0.6613	$< 10^{-4}$
cAP	cPH	0.8142	$< 10^{-4}$
cMPS	cMPD	0.9453	$< 10^{-4}$
cMPS	cPH	0.6937	$< 10^{-4}$
cMPS	SP	0.8758	$< 10^{-4}$
cMPS	PPP	0.6788	$< 10^{-4}$
cMPD	cSP	0.8224	$< 10^{-4}$
cMPD	PPP	0.5040	$< 10^{-4}$
cPH	cSP	0.6077	$< 10^{-4}$
cPH	PPP	0.8114	$< 10^{-4}$
cSP	PPP	0.8095	$< 10^{-4}$
